# Characterization and comparison of post-natal rat Achilles tendon-derived stem cells at different development stages

**DOI:** 10.1038/srep22946

**Published:** 2016-03-14

**Authors:** Jialin Chen, Wei Zhang, Zeyu Liu, Ting Zhu, Weiliang Shen, Jisheng Ran, Qiaomei Tang, Xiaonan Gong, Ludvig J. Backman, Xiao Chen, Xiaowen Chen, Feiqiu Wen, Hongwei Ouyang

**Affiliations:** 1 Centre for Stem Cell and Tissue Engineering, School of Medicine, Zhejiang University, Hangzhou, 310058, China; 2 Zhejiang Provincial Key Lab for tissue engineering and regenerative medicine, 310000, Hangzhou, China; 3 Department of Orthopaedics, Second Affiliated Hospital, Zhejiang University, Hangzhou, 310009, China; 4 Department of Integrative Medical Biology, Anatomy, Umeå University, Umeå, 90187, Sweden; 5 Division of haematology and oncology, Shenzhen Children’s Hospital, Shenzhen, 518038, China; 6 Department of Sports Medicine, School of Medicine, Zhejiang University, Hangzhou, 310058, China; 7 China Orthopedic Regenerative Medicine Group (CORMed), Hangzhou, 310058, China.

## Abstract

Tendon stem/progenitor cells (TSPCs) are a potential cell source for tendon tissue engineering. The striking morphological and structural changes of tendon tissue during development indicate the complexity of TSPCs at different stages. This study aims to characterize and compare post-natal rat Achilles tendon tissue and TSPCs at different stages of development. The tendon tissue showed distinct differences during development: the tissue structure became denser and more regular, the nuclei became spindle-shaped and the cell number decreased with time. TSPCs derived from 7 day Achilles tendon tissue showed the highest self-renewal ability, cell proliferation, and differentiation potential towards mesenchymal lineage, compared to TSPCs derived from 1 day and 56 day tissue. Microarray data showed up-regulation of several groups of genes in TSPCs derived from 7 day Achilles tendon tissue, which may account for the unique cell characteristics during this specific stage of development. Our results indicate that TSPCs derived from 7 day Achilles tendon tissue is a superior cell source as compared to TSPCs derived from 1 day and 56 day tissue, demonstrating the importance of choosing a suitable stem cell source for effective tendon tissue engineering and regeneration.

Tendons transmit forces from muscle to bone, maintaining joint motion and body movement. Tendon injuries are commonly encountered in the clinic, disrupting the patient’s working ability and spoiling the career of athletes. Injured tendons cannot heal adequately by themselves: injury often resulting in the formation of scar tissue. The risk of re-injury is in turn raised as a result of inferior mechanics of scar tissue^1^.

In recent years, tissue engineering approaches have been adopted by scientists to repair and regenerate the injured tendons using stem cell therapy^2^,^3^,^4^. Bone marrow mesenchymal stem cells (BMSCs) are the most widely used stem cells in tissue engineering, posing as a promising treatment options for damaged tissues such as bone and cartilage^5^,^6^. BMSCs have also been used in tendon tissue engineering for tendon repair. However, some studies have reported ectopic bone formation after treatment^7^ which contradicts the application of BMSCs in tendon repair. Tendon stem/progenitor cells (TSPCs) were first identified and described by Bi *et al.*^8^. These cells possess self-renewal ability and multi-differentiation potential to mesenchymal cells such as adipocytes, chondrocytes, and osteocytes. Recently, an array of studies^9^,^10^,^11^ have used TSPCs for tendon tissue engineering, which has shown promising potential for tendon repair and regeneration.

It has been reported that fetal dermal fibroblasts and adult dermal fibroblasts possess different tendon repair abilities^12^,^13^, indicating that cells isolated from different development stages harbour different regeneration potential. Tendon development is a process with striking changes of tissue structure and cell morphology^14^,^15^,^16^. Transforming growth factor beta (TGF-β) and mitogen-activated protein kinase (MAPK) signalling pathways have been found to be the two most strongly modified pathways during tendon development^17^. The signalling pathways and extracellular matrix (ECM) niches regulate the fate of TSPCs^8^,^10^,^18^, which in turn contribute to the homeostasis of tendon tissue. TSPCs in different development stages may have different potential for tendon regeneration. However this has not been elucidated yet.

On the basis of previous studies, we hypothesize that there will be a difference in self-renewal and multi-potent differentiation potential between TSPCs at different stages of development. Therefore, the aims of this study were to characterize and compare post-natal rat Achilles tendon tissue and TSPCs at different stages of development. To that end, we analysed the post-natal development process of Sprague-Dawley rat Achilles tendon tissue at different time-points by histology staining, TEM scanning and polarized light observation. TSPCs were evaluated at three different time-points (1, 7 and 56 days) and parameters such as clone formation ability, surface CD markers expression, differentiation potential to mesenchymal tissue, cell proliferation, and gene expression profile were compared.

## Results

### Morphological evaluation of rat Achilles tendon tissue

#### Haematoxylin and eosin (H&E) staining

**Figure 1:**
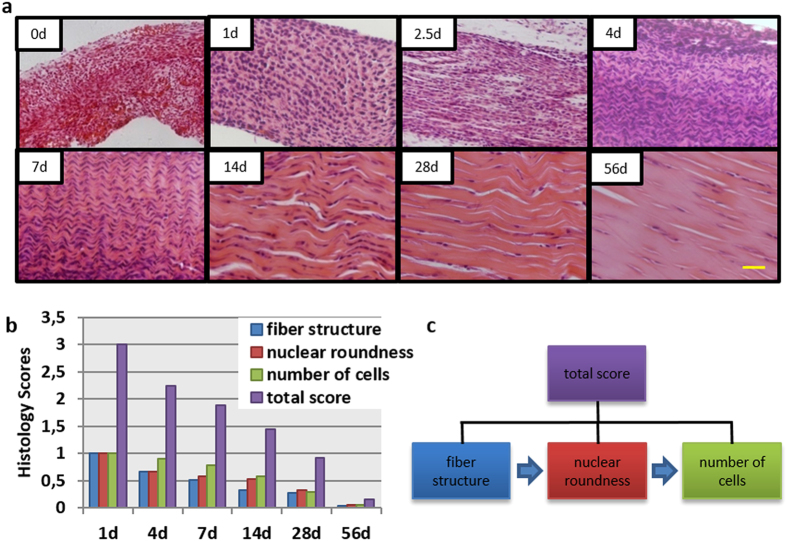
H&E staining and histology score evaluation of post-natal rat Achilles tendons. (**a**) H&E staining at day 0, 1, 2.5, 4, 7, 14, 28 and 56. Scale bars = 50 μm. (**b**) Histology score evaluation. Total score = fibre structure + nuclear roundness + number of cells. (**c**) Three parameters of histology score decreased in a distinct order during development.

Achilles tendons of post-natal rats were harvested at day 0, 1, 2.5, 4, 7, 14, 28 and 56. H&E staining revealed that the Achilles tendons experienced striking changes of tissue morphology (Fig. 1a), mainly on three parameters. Firstly, tissue structure became denser and more regular with deposition of new fibres with time. Obvious parallel and dense collagen fibres with short crimp pattern were found at day 7. Align tissue structure with long crimp pattern was observed at day 56. Secondly, the morphology of the nuclei turned from round shape at day 1, to short spindle shaped at day 7, and eventually to long spindle shaped at day 56. Thirdly, the number of cells decreased with development. The tendon tissue at day 56 contains only a small quantity of cells, which were surrounded by dense collagen fibres.

Histology score of the three above-mentioned parameters were compared at all time-points (Fig. 1b). The score of each parameter decreased with time; the lowest value being seen at day 56. Interestingly, it was found that the three parameters decreased in a specific order: first the fibre structure, then the nuclear roundness, and finally the number of cells (Fig. 1c).

#### Polarized light observation

**Figure 2:**
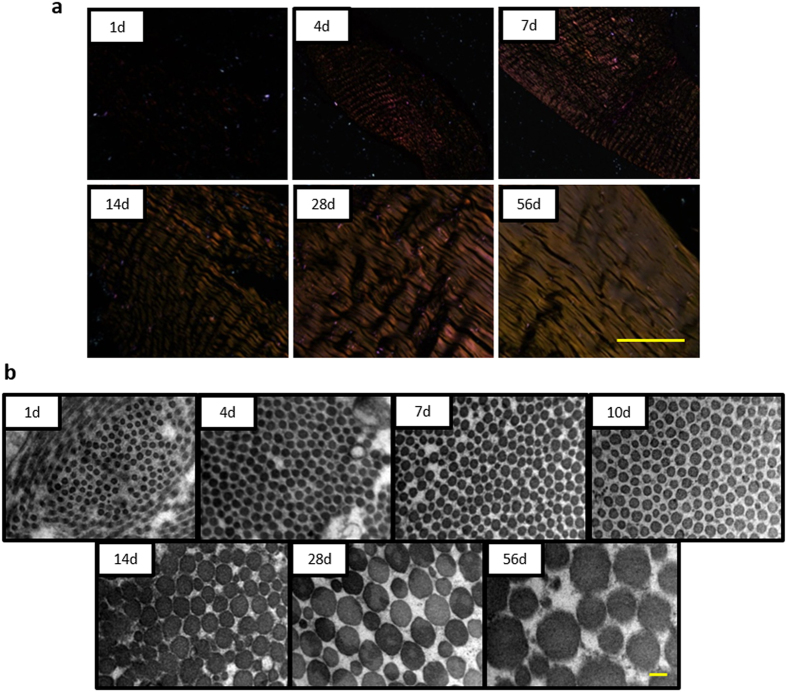
Maturation of tendon tissues during development. (**a**) Polarized light images at day 0, 4, 7, 14, 28 and 56. Scale bars = 200 μm. (**b**) Ultrastructure of tendon tissue by TEM at day 1, 4, 7, 10, 14, 28 and 56. Scale bars = 0.1 μm.

In order to evaluate the maturation level of tissue, histological sections were observed under polarized light microscopy (Fig. 2a). It was found that tendon tissue could not be observed at day 1, and partially observed at day 4. Notably, the main part of tendon tissue showed a weak golden yellow colour at day 7, i.e. the tissue structure is determined but the tissue maturation still needs to be developed. Parallel and golden yellow collagen fibres could be seen under the polarized light microscopy at day 56 tendon tissue.

#### Transmission electron microscopy (TEM) evaluation

To evaluate the change of collagen fibre diameter, transverse sections of tendon tissues were compared using TEM (Fig. 2b). It was found that the new-born tendon tissue consisted of small homogeneous collagen fibres (average diameter is 32.76 nm). The diameter of collagen fibres increased with development. At day 56, larger heterogeneous collagen fibres were formed (average diameter is 165.54 nm). It was noticed that a trend of difference in collagen fibre began at day 7 and two categories of collagen fibres with different diameters were obviously found at day 14.

### Comparison of TSPCs at different development stages

#### Isolation of TSPCs at different development stages

**Figure 3:**
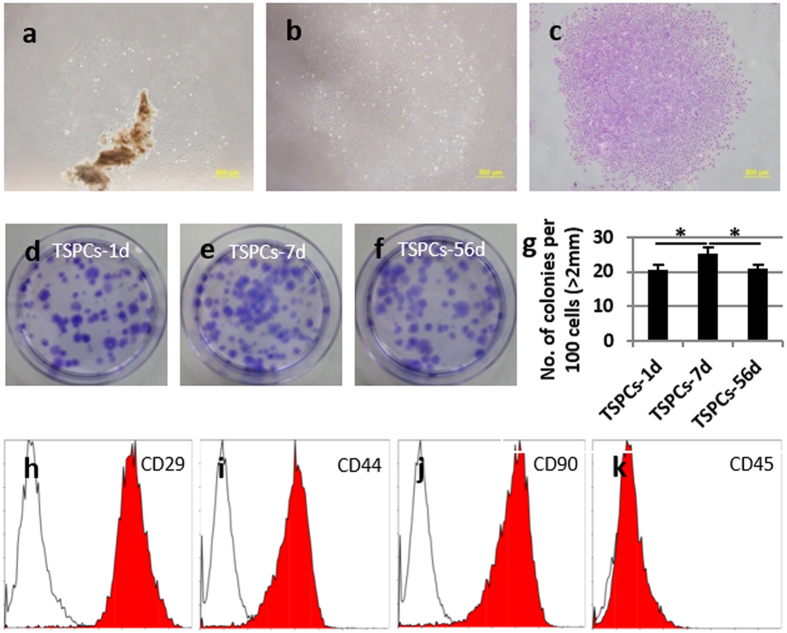
Isolation and characterization of TSPCs-1d, 7d and 56d. (**a**) Cells adhered and spread from tendon tissues. (**b**) Clone formed from single cell and (**c**) stained by crystal violet. (**d**–**g**) Clone formation ability comparison among different TSPCs. (**h**–**k**) Comparison of MSCs surface marker expression by flow cytometer. Scale bars = 500 μm (**a**–**c**). *Significant difference between two groups at p < 0.05.

According to the difference in tendon tissue development, we chose three representative time-points in which TSPCs were isolated: 1 day (TSPC-1d), 7 days (TSPC-7d) (tissue morphology experienced striking change around this time-point), and 56 days (TSPC-56d). It was found that the cells adhered and spread from tendon tissues (Fig. 3a). After low density screen, some single cell formed clones in dish (Fig. 3b,c).

#### Comparison of clone formation ability

The clone formation ability of TSPCs extracted from post-natal tendon tissue at day 1, 7 and 56 was compared by crystal violet staining (Fig. 3d–g). All TSPCs possessed a high ability to form clones, however TSPCs-7d (25.33 ± 1.73%) had a significantly higher ability to form clones as compared to TSPCs-1d (20.44 ± 1.71%, p < 0.05) and TSPCs-56d (21.00 ± 1.00%, p < 0.05).

#### Comparison of surface marker expression using fluorescence-activated cell sorting (FACS)

TSPCs derived from day 1, 7 and 56 tendon tissue showed similar expression profile of CD marker as MSCs (Fig. 3h–k). They all expressed CD29, CD44, and CD90 with no significant difference between the time-points (Table 1). All TSPCs were negative for hematopoietic marker CD45 (Fig. 3k).

#### Comparison of differentiation potential to mesenchymal tissue

**Figure 4:**
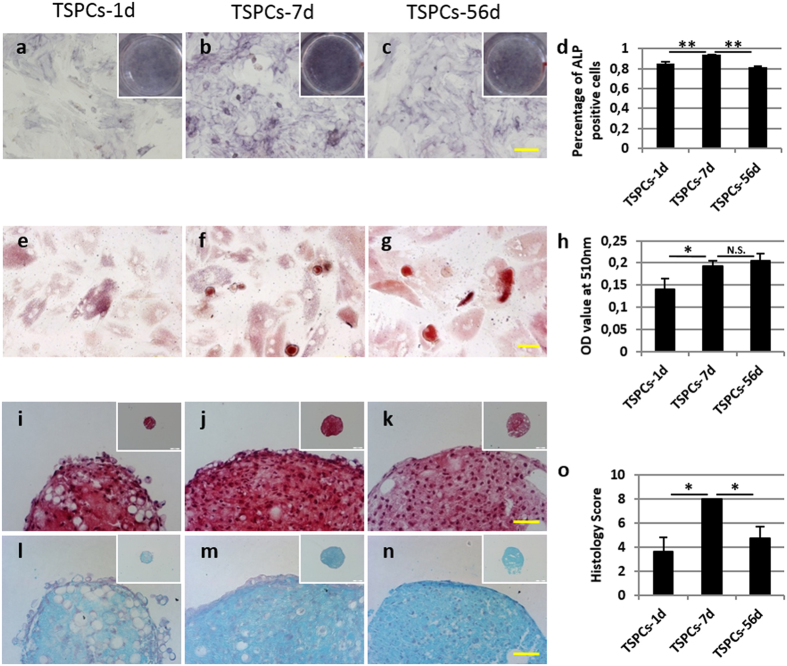
Comparison of mesenchymal lineage differentiation potential towards osteo-, adipo- and chondro-lineage among TSPCs derived from day 1, 7 and 56. (**a**–**d**) ALP staining for osteogenic differentiation after 14 days was performed and the percentages of positive cells were compared. (**e**–**h**) Oil red staining for adipogenic differentiation after 14 days was performed and quantified. (**i**–**o**) Safranin O staining and alcian blue staining for chondrogenic differentiation after 28 days were performed and histology scores were compared. Scale bars =100 μm (**a**–**c**), 50 μm (**e**–**g**,**i**–**n**) and 200 μm (insets of **i**–**n**). *Significant difference between two groups at p < 0.05. **Significant difference between two groups at p < 0.001. N.S. No significant difference between two groups at p ≥ 0.05.

The differentiation to mesenchymal tissue of TSPCs derived from day 1, 7 and 56 was compared. Osteogenic differentiation assay showed that TSPCs from all time-points could differentiate into osteo-lineage, as confirmed by ALP staining after two weeks induction (Fig. 4a–c). The percentage of ALP positive cells in TSPCs-7d group (93.82 ± 0.13%) was significantly higher than TSPCs-1d (85.21 ± 2.12%, p < 0.001) and TSPCs-56d (81.84 ± 0.81%, p < 0.001) (Fig. 4d). Oil red staining confirmed the adipogenic differentiation potential of TSPCs (Fig. 4e–g). It was found that the adipo-lineage differentiation ability of TSPCs-1d was weaker than TSPCs-7d (0.14 ± 0.02 vs. 0.19 ± 0.01, p < 0.05). However, there was no significant difference between TSPCs-7d and TSPCs-56d (p > 0.05) (Fig. 4h). Safranin O staining (Fig. 4i–k) and alcian blue staining (Fig. 4l–n) confirmed differentiation potential towards chondro-lineage phenotype. The histology score evaluation demonstrated that TSPCs-7d had stronger chondro-lineage differentiation ability as compared to TSPCs-1d (8.00 ± 0.00 vs. 3.67 ± 1.15, p < 0.05) and TSPCs-56d (8.00 ± 0.00 vs. 4.75 ± 0.96, p < 0.05) (Fig. 4o). Overall, TSPCs from all time-points possessed differentiation potential to osteo-, adipo- and chondro-lineage, however the differentiation ability of TSPCs-7d was stronger than TSPCs-1d and TPSCc-56d.

**Figure 5:**
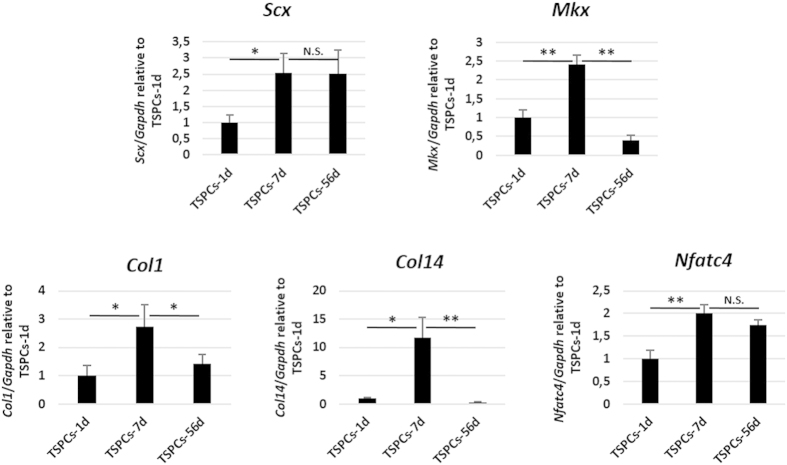
Comparison of teno-lineage differentiation potential among TSPCs derived from day 1, 7 and 56. The expression of tendon related genes *Scx*, *Mkx*, *Col1*, *Col14* and *Nfatc4* were compared by qPCR. *Significant difference between two groups at p < 0.05. **Significant difference between two groups at p < 0.001. N.S. No significant difference between two groups at p ≥ 0.05.

The tenogenic differentiation potential of TSPCs derived from day 1, 7 and 56 was compared in a cell-sheet model (Fig. 5). It was found that TSPCs-7d expressed higher *Scx* (p < 0.05), *Mkx* (p < 0.001), *Col1* (p < 0.05), *Col14* (p < 0.05) and *Nfatc4* (p < 0.001), compared to TSPCs-1d. There is no significant difference between TSPCs-7d and TSPCs-56d on the expression of *Scx* and *Nfatc4* (p > 0.05). However, much more expression of *Mkx* (p < 0.001), *Col1* (p < 0.05) and *Col14* (p < 0.001) was found in TSPCs-7d, compared to TSPCs-56d.

#### Comparison of proliferation ability

**Figure 6:**
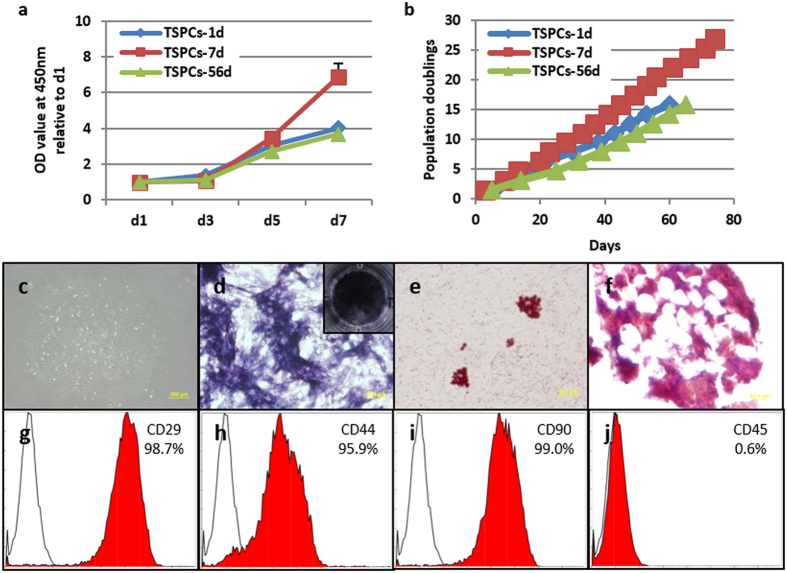
Comparison of cell proliferation ability among TSPCs-1d, 7d and 56d. (**a**) Short-term cell proliferation from 1–7 days in culture and (**b**) long-term population doublings of different TSPCs during a period of up to 80 days in culture. (**c**–**j**) Self-renewal and multi-potent differentiation potential of TSPCs-7d at passage 20. (**c**) Clone formation ability. (**d**) Osteogenic differentiation. (**e**) Adipogenic differentiation. (**f**) Chondrogenic differentiation. (**g**–**j**) MSCs surface maker expression. Scale bars =500 μm (**c**), 50 μm (**d**–**e**) and 20 μm (**f**).

The proliferation ability of TSPCs derived from day 1, 7 and 56 tendon tissue was compared at different days in culture. In short-term proliferation experiment (Fig. 6a), up to 3 days in culture, there was no significant difference between the TSPCs. From 5 days in culture, different OD value could be observed. At 7 days in culture, TSPCs-7d showed the highest OD value as compared to TSPCs-1d (6.90 ± 0.76 vs. 4.02 ± 0.11, p < 0.001) and TSPCs-56d (6.90 ± 0.76 vs. 3.68 ± 0.20, p < 0.001). A long-term proliferation experiment (Fig. 6b) showed that TSPCs-7d could proliferate to over 25 population doublings in 70 days in culture, which was higher and longer than for TSPCs-1d and TSPCs-56d. Most importantly, TSCPs-7d after 20 passages still possessed the ability to form clones (Fig. 6c), osteo-lineage differentiation potential (Fig. 6d), adipo-lineage differentiation potential (Fig. 6e), and some chondro-lineage differentiation potential (Fig. 6f), and showed similar expression profile of CD marker as MSCs (Fig. 6g–j).

#### Comparison of gene profile

**Figure 7:**
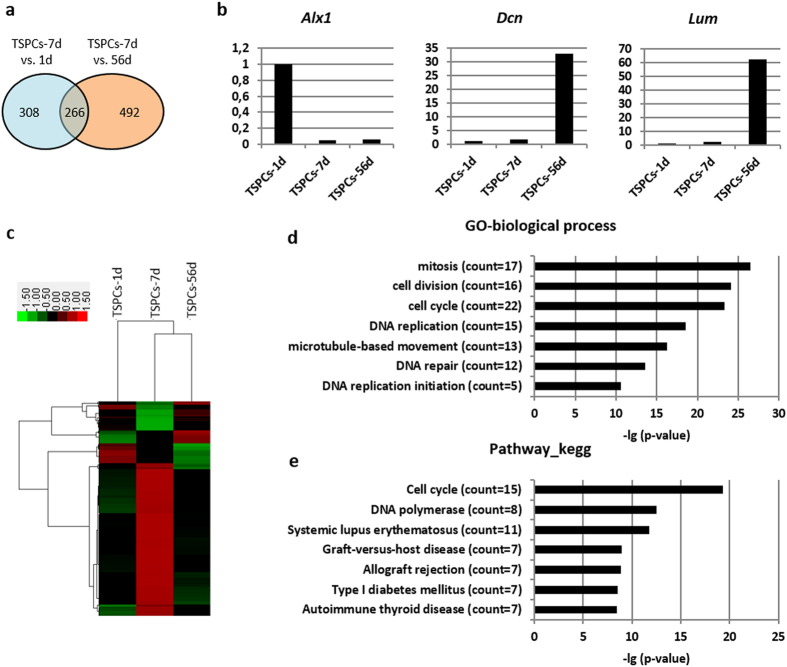
Microarray analysis of different TSPCs. (**a**) The number of transcripts with more than two-fold difference between TSPCs-7d and TSPCs-1d, or TSPCs-7d and TSPCs-56d. (**b**) qPCR results confirming differentially expression genes from microarray. (**c**) Gene cluster analysis of 266 transcripts, which showed more than two-fold difference both in TSPCs-7d vs. 1d, and TSPCs-7d vs. 56d. (**d**) Gene ontology related to biological process ordered according to –lg (p-value). (**e**) Gene ontology related to pathway ordered according to –lg (p-value). The array has been submitted to the GEO repository with accession number GSE68485 (http://www.ncbi.nlm.nih.gov/geo/query/acc.cgi?acc=GSE68485).

To elucidate the inherent molecular difference between TSPCs-7d and TSPCs-1 and TSPCs-56d, the gene profiles of each TSPCs were compared by microarray. A total of 1,066 transcripts out of 30,000 transcripts analysed were found to show a more than two-fold difference between TSPCs-7d and TSPCs-1d, or TSPCs-7d and TSPCs-56d (Fig. 7a). The expression of three randomly chosen genes (*Alx1*, *Dcn* and *Lum*) was confirmed by quantitative polymerase chain reaction (qPCR) (Fig. 7b). There were 266 transcripts showing more than two-fold difference both in TSPCs-7d vs. TSPCs-1d, and TSPCs-7d vs. TSPCs-56d, which may contribute to the different cell characteristics seen in this study (Supplementary Table S1, Fig. 7a). These 266 transcripts were hierarchically clustered. It was found that most of these genes were highly expressed in TSPCs-7d (Fig. 7c, red region in TSPCs-7d group). Gene Ontology (GO) analysis showed different biological processes (Fig. 7d) and pathways (Fig. 7e) between TSPCs-7d and TSPC-1d and 56d. In the biological process, the most significant GO term was ‘mitosis’ with 17 transcripts enriched, following by GO term related to cell division, cell cycle, DNA replication, microtubule-based movement, DNA repair and DNA replication initiation (Fig. 7d). Most of them were likely responsible for the high proliferation ability of TSPCs-7d. In the pathway related, the two most significant GO terms were ‘cell cycle’ and ‘DNA polymerase’ (Fig. 7e). The genes belonging to these GO terms were screened and listed in Supplementary Table S2. It was unexpectedly found that nearly all of the genes (52 out of 55 genes, 94.5%) were up-regulated in TSPCs-7d as compared to TSPCs-1d and 56d, which may account for the unique cell characteristics of TSPCs-7d during development.

## Discussion

This study establishes the first evaluation of tissue structure and morphological changes in post-natal rat Achilles tendon. Based on the results of tissue development, TSPCs from three different development stages (TSPC-1d, 7d and 56d) were isolated and compared. TSPCs-7d showed the highest self-renewal ability, cell proliferation, and differentiation potential towards mesenchymal lineage. These findings indicate that TSPCs-7d may be a better cell source than TSPCs-1d and TSPCs-56d for future cell therapy.

TSPCs have been studied for tendon tissue engineering and tendon repair since it was first discovered^8^,^9^,^19^. Similar as BMSCs, TSPCs possess self-renewal ability and multi-differentiation potential towards mesenchymal lineage. Moreover, these tissue specific stem cells express important tendon-related genes such as scleraxis and tenomodulin, and could form tendon tissues after implantation *in vivo*^8^. Therefore it is worth to further decipher the relation between TSPCs and tendon homeostasis.

There are some studies concerning the difference between young TSPCs and aged TSPCs, or young tendon tissues and aged tendon tissues. In 2003, Beredjiklian *et al.* found fetal sheep tendon to be able to regenerate itself whereas adult tendon could not. Collagen architecture was reconstituted in fetal tendon without any scar formation^20^. The same group further demonstrated that the regeneration ability of fetal tendon is dependent on intrinsic factors of the fetal tissue itself, rather than on environmental influence^21^. A similar scar-free repair phenomenon has been found in other tissues, such as skin^22^,^23^, nerve^24^ and bone^25^. The inherent mechanism remains unclear. As cells play an important role for tissue formation and tissue repair, and as TSPCs is the only stem cell that has been found in tendon tissue, it would be TSPCs that contributes to the different repair ability between fetal and adult tendons. Zhou *et al.* compared TSPCs derived from young rats (3–4 month) and old rats (24–26 month)^26^. They found that young TSPCs had a stronger proliferation and clone formation ability than old. Furthermore, the cell cycle progression of aged TSPCs was delayed and the differentiation potential to adipo-lineage was enhanced^26^. A recent study used TSPCs derived from young (28 ± 5 years) and healthy human tendons, and those derived from aged (63 ± 14 years) and degenerated human tendons^27^. It was found that aged TSPCs exhibited deficit in self-renewal and clone formation ability, with an elevation of senescence. However, the multi-lineage differentiation potency did not show any difference between young and aged TSPCs^27^. Similar as TSPCs, some studies found young BMSCs to have more clone formation and proliferated faster than old BMSCs^28^,^29^,^30^,^31^. All of these studies indicate that the function and fate of TSPCs is age dependent with the development or degeneration of tendon tissues, and young TSPCs has the potential to regenerate injured tendon without scar formation. However, tendon tissue experiences striking morphological and structural changes during development^14^,^15^,^16^,^32^ and it has been reported that tendon extracellular matrix regulates teno-lineage differentiation of TSPCs^10^,^33^,^34^. To better understand the intrinsic repair ability of young TSPCs, it is necessary to evaluate TSPCs in a more accurate time window with more detailed analysis during the tendon tissue development.

In the current study, we compared the difference in tissue structure and morphology of post-natal rat Achilles tendons at different time-points. It was shown, via H&E staining, that striking changes of tissue structure and morphology was observed over time. Collagen fibres deposited with time and they became more and more organized in their arrangement, which might be the reason for the spindle-shaped cell morphology and that the cell number decreased, as illustrated with the histology score evaluation. Polarized light observation and TEM evaluation further revealed the maturation process of tendon tissues. These results were consistent with previous studies in mice and rabbit models^14^,^15^,^16^,^32^. We chose the 7 days as an important time-point to study the TSPCs based on the finding that the crimp structure of tendon is already formed but not matured around this time point, as seen with H&E staining. This finding was further confirmed under polarized light observation, in which the whole tissue structure could be observed in 7 day tissue, which was not the case with the 1 day tissue. However, 7 day tissue showed only weak golden yellow colour as compared to 56 day. The 7 day time-point was also chosen as the nuclear morphology turned from round to short spindle-shaped at 7 days and as the diameters of collagen fibres began to be heterogeneous in the 7 to 14 day tissue.

TSPCs-7d had significantly higher proliferation ability and clone formation potential as compared to TSPCs-56d. However, it was interesting that there was not much difference between TSPCs-1d and TSPCs-56d in terms of proliferation. The advantage in cell proliferation ability of TSPCs-7d could be explained by up-regulated gene expression in GO terms related to mitosis, cell division, cell cycle in biological process and cell cycle, DNA polymerase in pathway. However, this needs to be confirmed in future studies. In addition, TSPCs-7d also had advantages in the differentiation potential towards mesenchymal lineage, notably to teno-lineage in cell-sheet model, making it as a candidate of ideal cell source for tendon tissue engineering. To further find crucial genes or factors that contribute to the formation of the optimal conditions of TSPCs-7d, it is valuable to study the molecular network and microenvironment in 1 day or earlier tendon tissue, which triggers and determines the cell fate transition from TSPCs-1d to TSPCs-7d (Chen *et al.*, unpublished). This information could contribute a lot to effectively stepwise tenogenic differentiation from stem cells to mature tenocytes, and thus promote tendon regeneration in the future.

In summary, TSPCs have different self-renewal and mesenchymal differentiation ability during tendon development. TSPCs-7d was found to have advantages in cell proliferation, clone formation, mesenchymal-lineage differentiation potential as compared to TSPCs-1d and TSPCs-56d. This study is not only helpful in understanding the biological roles of TSPCs during tendon development, but also provides valuable insight in suitable stem cell selection and stepwise tenogenic differentiation for future tendon tissue engineering.

## Methods

### Ethics statement

All studies were approved by the Zhejiang University Administration on Laboratory Animal Care. Animals were treated in accordance with IACUC guidelines (ethics approval number: ZJU2010102007).

### H&E staining

The harvested specimens (n ≥ 3 at each time point) were fixed in 10% (v/v) neutral buffered formalin, dehydrated through an alcohol gradient, cleared, and embedded in paraffin blocks. A microtome was used to prepare histological sections (7 *μ*m). These sections were stained with haematoxylin and eosin as explained previously^35^ and also observed under polarized light microscopy. Histological scoring^35^ was performed using a blinded semi-quantitative scoring system based on three parameters: fibre structure, nuclear roundness and number of cells. The score of each parameter in the rat Achilles tendon tissue from day 1 was set as 1.

### TEM

Tissue specimens were fixed by standard procedures for TEM to assess collagen fibril diameter^36^. Briefly, specimens were pre-fixed with 2% glutaraldehyde for 24 h and washed twice with phosphate-buffered saline (PBS) followed by post-fixation with 1% osmic acid for 2 h. After two washes in PBS, the specimens were dehydrated in an ethanol gradient and dried to a critical point. Subsequently, the specimens were sectioned transversely, mounted and sputter-coated with gold before analysed using TEM (Quanta 10 FEI). The average fibril diameter was calculated to reflect the development of collagen fibril.

### Cell isolation and culture

Rat Achilles tendon tissues from three different post-natal stages: 1 day, 7 days and 56 days were obtained following the approved guidelines set by Zhejiang University Institutional Animal Care. To isolate TSPCs at each development time point, tendon tissues from at least two individuals were mixed, cut into 1–2 mm^3^ pieces, and washed three times with PBS. The tissue fragments were cultured in low glucose DMEM (Gibco) supplemented with 10% (v/v) fetal bovine serum (Invitrogen) and 1% penicillin-streptomycin (Gibco). When cells had migrated from the tissue and adhered to the culture surface, they were trypsinized and seeded at very low density (2 cells/cm^2^) to form colonies. After 10–12 days, the colonies formed were trypsinized and passaged. All TSPCs in this study were of polyclonal origin. The cells were trypsinized when confluent and split 1:4. Cells were used between passages 3 and 5.

### Crystal violet staining

The colonies formed by TSPCs were stained with 1% crystal violet (Sigma) in methanol for 10 min. The number and size of all colonies with diameters >2 mm were counted.

### FACS analysis

Cells in suspension (5 × 10^5^) were incubated with 1 mg of FITC-conjugated mouse specific to rat monoclonal antibodies towards CD29, CD44, CD45 and CD90 (all from BD Pharmingen) for 1 h at 4 °C. FITC-conjugated isotype-matched IgGs (BD Pharmingen) were used as controls. After washing, the cells were incubated with FITC-conjugated rabbit anti-mouse IgG for 45 min on ice. After additional washing, the samples were analysed using a Coulter Epics XL flow cytometer.

### Evaluation of mesenchymal lineage differentiation potential of TSPCs

The multipotent differentiation potential of TSPCs toward adipogenic, osteogenic, and chondrogenic lineages was evaluated *in vitro* according to established protocols^19^. Positive induction of adipogenesis was confirmed by Oil Red O staining and the amount of extracted dye at 510 nm was determined (n = 3). Positive induction of osteogenesis was confirmed by alkaline phosphatase staining (n = 3). The count of ALP positive cells and total cells were counted and the ratio was compared between TSPCs-7d and TSPCs-1d, or TSPCs-7d and TSPCs-56d. Positive induction of chondrogenesis was confirmed by Safranin O staining (n = 3). Histology evaluation of each sample includes uniformity and intensity of Safranin O stain, distance between cells/amount of matrix produced, and cell morphology, with a score ranging from 0 to 3. The sum of histology score was calculated and compared between different groups.

The differentiation potential of TSPCs toward teno-lineage was evaluated in an *in vitr*o cell-sheet differentiation model. As described previously^37^, upon confluence, TSPCs were cultured in DMEM supplemented with 10% (v/v) fetal bovine serum and 50 μg/ml ascorbic acid (Sigma). Samples after 3 days culture were collected for mRNA extraction and following qPCR evaluation.

### Cell proliferation assay and population doubling

Cell proliferation was measured with the Cell Counting KIT-8 (CCK-8, Dojindo) and following the manufacturer’s protocol. The cells were cultured for 1, 3, 5 and 7 days and incubated with CCK-8 solution in a 5% CO_2_ incubator at 37 °C for 2 h. The amount of formazan produced was measured at 450 nm using a spectrophotometer.

Population doubling at each passage was calculated using the equation: n = [log (final cell count) − log (number of cells initially plated)]/0.301. Cumulative population doubling was obtained by adding population doubling to the previous one^38^.

### Microarray assay

TSPCs at different post-natal development stages (1d, 7d and 56d) were isolated from at least two individuals. They were cultured and used for microarray analyses at passage 2. Total RNA was extracted using Trizol reagent and was further purified using Qiagen RNeasy Mini Kit according to the manufactures’ instructions. The RNA quality was assessed by formaldehyde agarose gel electrophoresis. An aliquot of 200 ng of total RNA was used to synthesize double-stranded cDNA, and then produce biotin-tagged aRNA using MessageAmp™ Premier RNA Amplification Kit (Life Technologies). The resulting bio-tagged aRNA was fragmented to strands of 35–200 bases in length according to the protocols from Affymetrix. The fragmented aRNA was hybridized to Rat Genome 230 2.0 Array (Affymetrix) containing 30,000 transcripts. Hybridization was performed at 45 °C with rotation for 16 h at hybridization oven 640. The GeneChip arrays were washed and then stained automatically on an Affymetrix Fluidics Station 450 followed by scanning on an Affymetrix GeneChip Scanner 3000 7G.

The scanned images were first assessed by visual inspection then analysed to generate raw data files saved as CEL files using the default setting of Affymetrix^®^ GeneChip^®^ Command Console^®^3.2 (AGCC) Software. Then the raw data were normalized and summarized with the Affymetrix Microarray Suite 5.0 (MAS5) and with the Robust Multi-array Average (RMA) algorithm^39^. Fold change was the ratio of two normalized sample signal. Genes were determined to be significantly differentially expressed using fold change of 2.0 as cutoffs. Hierarchical clustering with the average linkage method was performed with Cluster3.0 software and the cluster result was visualized through with the Treeview program. The array has been submitted to the GEO repository with accession number GSE68485.

### RNA isolation and qPCR

Total mRNA was isolated by cellular lysis in Trizol (Invitrogen) followed by a one-step phenol chloroform-isoamyl alcohol extraction, according to the manufacturer’s instructions. qPCR was performed to compare the expression of genes using SYBR Green qPCR Master Mix (TakaRa) with a Light Cycler apparatus (ABI 7900HT), as described previously^40^. The results are presented as target gene expression normalized to GAPDH.

### Statistical analysis

All quantitative data are presented as mean ± SD. One-way ANOVA and Bonferroni post hoc test were performed to assess statistical significance of results between groups. Values of p < 0.05 were accepted as statistically significant.

## Additional Information

**How to cite this article**: Chen, J. *et al.* Characterization and comparison of post-natal rat Achilles tendon-derived stem cells at different development stages. *Sci. Rep.* **6**, 22946; doi: 10.1038/srep22946 (2016).

## Supplementary Material

Supplementary Information

## Figures and Tables

**Table 1:** MSCs surface maker expression of TSPCs-1d, 7d and 56d.

	TSPCs-1d	TSPCs-7d	TSPCs-56d
CD29	95.8%	99.1%	92.9%
CD44	86.4%	93.7%	76.3%
CD90	92.5%	97.7%	96.4%
CD45	0.1%	0.1%	0.1%
