# Supplementary material for: Characterization and comparison of post-natal rat Achilles tendon-derived stem cells at different development stages
Source: Sci Rep. 2016 Mar 14;6:22946. doi: 10.1038/srep22946 (PMC4789738; doi:10.1038/srep22946)
Supplement: Supplementary Information [file srep22946-s1.pdf]

Characterization and comparison of post-natal rat Achilles tendon-derived stem cells at different development stages

Jialin Chen, Wei Zhang, Zeyu Liu, Ting Zhu, Weiliang Shen, Jisheng Ran, Qiaomei Tang, Xiaonan Gong, Ludvig J. Backman, Xiao Chen, Xiaowen Chen, Feiqiu Wen, Hongwei Ouyang

Supplemental table S1. Differentially regulated gene numbers between every two TSPCs.

Number of genes	TSPCs-7d vs. 1d	TSPCs-7d vs. 56d	TSPCs-56d vs. 1d
Up regulated	359(63%)	439(58%)	376(46%)
Down regulated	215(37%)	319(42%)	446(54%)
Differentially regulated	574	758	822

Supplemental table S2. Genes screened from top seven biological process GO terms and top two pathway GO terms.

ProbeSetID	Gene symbol	Gene name	Fold change<br><br>TSPCs-7d vs.1d	Fold change<br><br>TSPCs-7d vs.56d
1368260_at	*Aurkb*	aurora kinase B	2.69	2.73
1368799_at	*Birc5*	baculoviralIAP repeat-containing 5	2.12	2.65
1379582_a_at	*Ccna2*	cyclin A2	2.90	3.52
1370345_at	*Ccnb1*	cyclin B1	2.64	2.70
1370346_at			3.30	2.78
1389566_at	*Ccnb2*	cyclin B2	2.79	3.18
1367776_at	*Cdc2*	cell division cycle 2, G1 to S and G2 to M	2.26	2.38
1370294_a_at	*Cdc20*	cell division cycle 20 homolog (S. cerevisiae)	2.61	2.79
1389506_x_at			2.13	2.83
1379234_a_at	*Cdc45l*	CDC45 cell division cycle 45-like (S. cerevisiae)	2.72	2.00

1374449_at	*Cdca3*	cell division cycle associated 3	2.96	2.28
1371928_at	*Cdca8*	cell division cycle associated 8	2.22	2.58
1389542_at	*Cenpe*	centromere protein E	2.80	3.18
1373823_at	*Cks2*	CDC28 protein kinase regulatory subunit 2	2.33	2.36
1397704_at	*Esco2*	establishment of cohesion 1 homolog 2 (S. cerevisiae)	3.31	2.64
	LOC685024	similar to N-acetyltransferase ESCO2 (Establishment of cohesion 1 homolog 2) (ECO1 homolog 2)		
	LOC691979	similar to N-acetyltransferase ESCO2 (Establishment of cohesion 1 homolog 2) (ECO1 homolog 2)		
1385733_at	*Exo1*	exonuclease 1	2.80	2.39
1378240_at	*Fancd2*	Fanconianemia, complementation group D2	2.83	2.71
1389103_at	*Gins4*	GINS complex subunit 4 (Sld5 homolog)	2.17	2.08
1390891_at	*Kif11*	kinesin family member 11	2.81	2.96

1374794_at	*Kif15*	kinesin family member 15	2.69	3.30
1372903_at	*Kif18b*	kinesin family member 18B	2.42	2.97
1373722_at	*Kif20a*	kinesin family member 20A	2.36	2.89
1380775_at	*Kif20b*	kinesin family member 20B	2.72	3.62
1372516_at	*Kif22*	kinesin family member 22	2.50	2.36
1391063_at	*Kif23*	kinesin family member 23	2.47	2.63
1374912_at	*Kif2c*	kinesin family member 2C	2.75	2.41
1379479_at	*Kif4*	kinesin family member 4	3.51	2.97
1376185_at	*Kifc1*	kinesin family member C1	2.88	2.56
1368204_at	*Lig1*	ligase I, DNA, ATP-dependent	2.44	2.34
1379195_s_at			2.18	2.12
1376346_at	*LOC681162*	similar to Cyclin-dependent kinases regulatory subunit 1 (CKS-1) (Sid 1334)	0.32	0.34
	RGD1561797	RGD1561797		

1376951_at	*Mad2l1*	MAD2 (mitotic arrest deficient, homolog)-like 1 (yeast)	2.79	2.88
1378028_at			2.54	2.56
1398602_at			2.73	2.58
1376055_at	*Mcm5*	minichromosome maintenance complex component 5	3.02	2.36
1371074_a_at	*Mcm6*	minichromosome maintenance complex component 6	3.35	2.12
1388744_at	*Mcm7*	minichromosome maintenance deficient 7 (S. cerevisiae)	3.07	2.39
1381130_at	*Mcm8*	minichromosome maintenance complex component 8	2.92	2.60
1377299_at	*Nasp*	nuclearautoantigenic sperm protein (histone-binding)	2.46	2.66
1378264_at			2.02	2.30
1374799_at	*Ncapd2*	non-SMC condensin I complex, subunit D2	2.58	3.28
1378296_at	*Ncaph*	non-SMC condensin I complex, subunit H	3.14	2.89
1383940_at	*Nuf2*	NUF2, NDC80 kinetochore complex component, homolog (S. cerevisiae)	2.66	2.79
1385522_at	*Orc1l*	origin recognition complex, subunit 1-like (yeast)	3.25	2.26

1370297_at	*Plk1*	polo-like kinase 1 (Drosophila)	2.66	2.57
1376611_at	*Pola1*	polymerase (DNA directed), alpha 1	2.38	2.38
1389421_at	*Pole*	polymerase (DNA directed), epsilon	2.06	2.66
1383578_at	*Rad51*	RAD51 homolog (RecA homolog, E. coli) (S. cerevisiae)	3.39	2.64
1389326_at	*Rfc3*	replication factor C (activator 1) 3	2.09	2.17
1388458_at	*Rfc4*	replication factor C (activator 1) 4	2.12	2.44
1395944_at	*RGD1310778*	similar to Putative protein C21orf45	2.50	2.60
1368144_at	*Rgs2*	regulator of G-protein signaling 2	0.40	2.15
1393848_at	*Rrm2*	ribonucleotidereductase M2	3.03	2.47
1389668_at	*Spc25*	SPC25, NDC80 kinetochore complex component, homolog (S. cerevisiae)	3.05	2.70
1368522_at	*Timeless*	timeless homolog (Drosophila)	2.41	2.33
1372631_at	*Tk1*	thymidine kinase 1, soluble	2.93	2.30
1378640_at	*Uhrf1*	ubiquitin-like with PHD and ring finger domains 1	3.49	2.78

1373538_at	*Usp1*	ubiquitin specific peptidase 1	2.86	2.81
1376687_at			2.93	2.39
